# DNA Testing Reveals the Putative Identity of JB55, a 19th Century Vampire Buried in Griswold, Connecticut

**DOI:** 10.3390/genes10090636

**Published:** 2019-08-22

**Authors:** Jennifer Daniels-Higginbotham, Erin M. Gorden, Stephanie K. Farmer, Brian Spatola, Franklin Damann, Nicholas Bellantoni, Katie S. Gagnon, Maria de la Puente, Catarina Xavier, Susan Walsh, Walther Parson, Timothy P. McMahon, Charla Marshall

**Affiliations:** 1 Armed Forces Medical Examiner System’s Armed Forces DNA Identification Laboratory (AFMES-AFDIL), Dover Air Force Base, Dover, DE 19902, USA; 2 SNA International (Contractor Supporting the AFMES-AFDIL), Alexandria, VA 22314, USA,; 3 Department of Biology – Forensic and Investigative Sciences Program, Indiana University-Purdue University Indianapolis, Indianapolis, IN 46202, USA; 4 National Museum of Health and Medicine, Anatomical Division, Defense Health Agency, Silver Spring, MD 20910, USA; 5 Defense POW/MIA Accounting Agency, Offutt Air Force Base, Sarpy County, NE 68113, USA; 6 Department of Anthropology, University of Connecticut, Storrs, CT 06269, USA; 7 Independent Researcher, Plainville, CT 06062, USA; 8 Forensic Genetics Unit, Institute of Forensic Sciences, University of Santiago de Compostela, 15782 Santiago de Compostela, Spain; 9 Institute of Legal Medicine, Medical University of Innsbruck, 6020 Innsbruck, Austria; 10 Forensic Science Program, The Pennsylvania State University, University Park, PA 16802, USA

**Keywords:** vampire, surname prediction, ancestry estimation, historical archaeology, Next-Generation Sequencing, DNA identification, SNP, Y-STR, genetic genealogy, tuberculosis

## Abstract

In 1990 in Griswold, Connecticut, archaeologists excavated a burial found in a “skull and crossbones” orientation. The lid of the 19th century coffin had brass tacks that spelled “JB55”, the initials of the person lying there and age at death. JB55 had evidence of chronic pulmonary infection, perhaps tuberculosis. It is possible that JB55 was deemed a vampire due to his disease, and therefore had to be “killed” by mutilating his corpse. In an attempt to reveal the identity of JB55, DNA testing was performed. Ancestry informative single nucleotide polymorphism (SNP) analysis using the Precision ID Ancestry Panel indicated European ancestry. A full Y-chromosomal short tandem repeat (Y-STR) profile was obtained, belonging to haplogroup R1b. When the Y-STR profile was searched in the publicly accessible FamilyTreeDNA R1b Project website, the two closest matches had the surname “Barber”. A search of historical records led to a death notice mentioning John Barber, whose son Nathan Barber was buried in Griswold in 1826. The description of Nathan Barber closely fits the burial of “NB13,” found near JB55. By applying modern forensic DNA tools to a historical mystery, the identity of JB55 as John Barber, the 19th century Connecticut vampire, has been revealed.

## 1. Introduction

**Figure 1:**
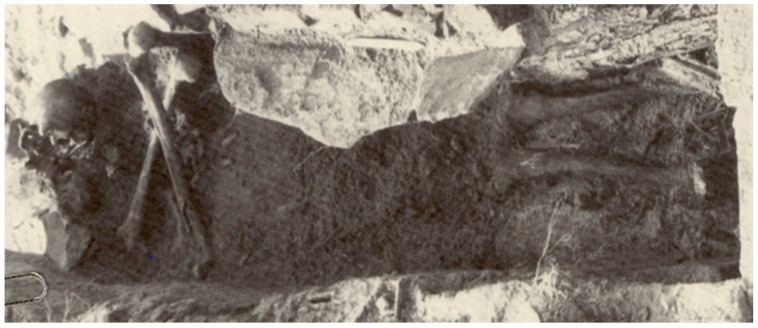
Photograph of JB55 showing the long bones arranged in an “X” directly under the skull in a “skull and cross bones” orientation.

**Figure 2:**
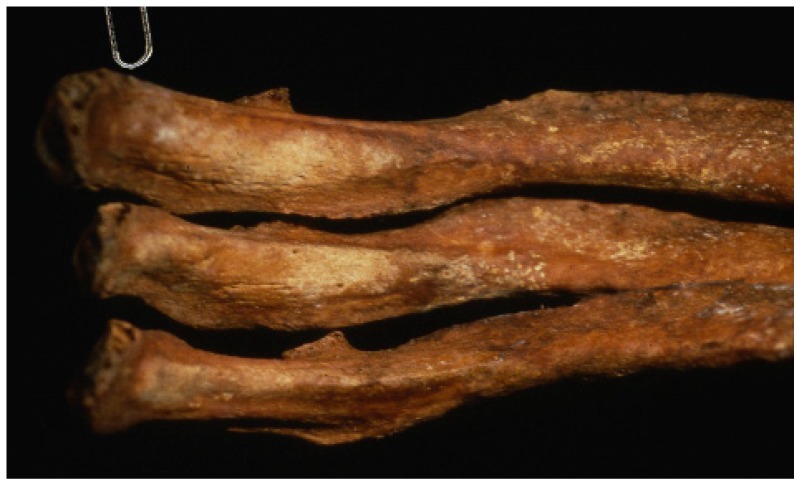
The ribs of JB55 showing pathological lesions consistent with chronic lung infection, perhaps caused by tuberculosis.

In 1990, an unmarked cemetery dating to the 18th–19th centuries was excavated in Griswold, Connecticut, when skeletal remains were encountered during sand and gravel operations [1]. Of the twenty-seven burials discovered, a stone-lined grave containing a middle-aged male proved to be very interesting. Brass tacks on the coffin lid spelled “JB55”, likely indicating the initials of the deceased and the age at death of 55 years. The remaining hardware included screws and copper dowel hinges, which dated the coffin to the early nineteenth century [1]. Most notably, the skull and femora of JB55 were found in a "skull and crossbones" orientation (Figure 1), indicating postmortem rearrangement of the remains. Additionally, JB55 displayed evidence of chronic lung infection in the form of proliferative lesions on the pleural surfaces of the ribs (Figure 2) [2,3]. This lung infection may have been tuberculosis (TB), a highly contagious disease caused by the *Mycobacterium tuberculosis* pathogen that was prevalent in the 1800s before antibiotics became available. The side effects of TB include jaundice (pale and yellow skin), red and swollen eyes, the presence of blood around the mouth from coughing, and the overall appearance of “wasting away”, all of which align with the physical attributes commonly associated with vampires [1,4,5]. 

A vampire belief system was circulating Griswold and its borough Jewett City during the mid-1800s. The famous Jewett City vampires were a large farming family that lost multiple male family members over nine years to tuberculosis or “consumption”. When another young son was stricken with the illness, the family became convinced they were plagued by vampires. Therefore, they disinterred the dead, burned and reburied their remains. The young boy recovered and they took this as a sign that the practice worked [5,6]. Such vampire folklore attributed the high number of deaths resulting from disease to vampires rising from the dead and feeding on living relatives. In attempts to stop the vampire “epidemic”, the body of a diseased individual was often exhumed and examined. The presence of certain characteristics (e.g., blood draining from the mouth and a bloated chest), while now known to be associated with the natural process of decomposition, were mistaken for indications of life [4,6]. In order to kill the vampire, the vital organs of the decedent were often burned, including most notably the heart. When no organs were present, a common practice involved the separation of the skull from the body [4]. As explained in their 1994 paper describing the JB55 burial, Sledzik and Bellantoni hypothesized “that, in the absence of a heart to be burned, the apotropaic remedy was to place the bones in a “skull and crossbones” arrangement. In support of this hypothesis, [the authors] note that decapitation was a common European method of dispatching the dead vampire, and that the Celts and Neolithic Egyptians were known to separate the head from the body, supposedly to prevent the dead from doing harm [citing Barber 1988]” [4]. Based on pathological evidence and knowledge of local vampire beliefs and burial practices, the totality of the evidence suggests that JB55 may have died of TB and was treated as a vampire.

Samples from the remains of JB55 and other burials from the cemetery site were sent to the National Museum of Health and Medicine (NMHM) in the early 1990s for curation and future scientific investigation. At that time, a sample from the femur was sent to the Armed Forces DNA Identification Laboratory (AFDIL, a branch of the Armed Forces Medical Examiner System (AFMES-AFDIL)) for DNA testing. However, methods available at the time provided only limited information from historical samples, such as mitochondrial DNA (mtDNA) control region sequence data. Since mtDNA is maternally inherited and does not undergo recombination, it can be used as a maternal lineage marker for DNA-assisted identification. Yet in the absence of known maternal relatives for mtDNA sequence comparison, the identification of JB55 was not possible. Today, advances in DNA technology make it possible to learn more from ancient and historic burials than ever before. Single nucleotide polymorphisms (SNPs) can provide valuable information on individual ancestry, as can haplogrouping of haploid markers. Additionally, the analysis of short tandem repeats (STRs) in the Y-chromosome may enable surname prediction of an unknown individual [7]. The goal of the present study was to apply current DNA techniques in an attempt to reveal the identity of JB55. This report exemplifies the strength of genomic technology in settling a decades-old historical mystery, that of the Griswold, Connecticut vampire.

## 2. Materials and Methods 

### 2.1. Contamination Prevention

The laboratory work was performed at the AFMES-AFDIL, an ISO 17025 accredited forensic DNA testing laboratory. The lab is divided into designated spaces for: (1) sample preparation and bone powdering; (2) DNA extraction, library preparation, and PCR setup; and (3) post-PCR manipulation and sequencing. The clean laboratories are supplied with positive pressure and are decontaminated with bleach on a regular basis. The post-amplification laboratories have negative air pressure to contain amplified product. Standard precautionary measures are taken to prevent contamination of the sample with exogenous DNA. These include the use of double gloves, sleeve guards, disposable personal protective equipment, molecular grade reagents, and UV-irradiated consumables. 

### 2.2. DNA Extraction

Two independent DNA extractions were completed from approximately 500 mg each of femoral bone powder. An extraction reagent blank (RB) was generated for each extraction and processed simultaneously. The bone was demineralized overnight at 56 °C in 7.5 mL of 0.5 M EDTA with 1% N-Lauryl sarcosine and 200 μL of 20 mg/mL proteinase K (Thermo Fisher Scientific, Waltham, MA, USA) [8]. Following complete demineralization, an organic extraction was performed. This involved two equi-volume washes with phenol chloroform isoamyl alcohol (PCIA) followed by centrifugation for 3 minutes at 4000× g. The upper aqueous layer was transferred to an Ultra-4/10 KDa filter (Millipore Sigma, Burlington, MA, USA) for buffer exchange. After the sample was concentrated to 500 µL by centrifugation at 5,000× g, two washes were completed using 2 mL 10 mM Tris-HCl (pH 7.5). Quantity sufficient (qs) elution buffer (Tris-EDTA: 10 mM Tris-HCl, 0.1 mM EDTA, pH 7.5) was added to bring the final sample volume up to approximately 200 μL. 

### 2.3. DNA Repair and Purification

DNA extracts and associated RBs were treated with the NEBNext FFPE DNA Repair Mix (New England BioLabs, Ipswich, MA, USA) following the manufacturer’s recommended protocol. This enzymatic DNA repair step has been shown to improve PCR amplification of DNA extracted from historical bone samples [9]. Repaired samples were purified using the QIAGEN MinElute PCR purification kit (QIAGEN, Hilden, Germany). DNA was eluted in 53 μL of sterile Tris-EDTA.

### 2.4. DNA Quantification 

Quantification of human DNA was completed using the Plexor HY DNA Quantification Kit (Promega Corporation, Madison, WI, USA) following the manufacturer’s protocol. The human DNA concentration was used to determine input volume into the SNP and Y-chromosomal short tandem repeat (Y-STR) assays. 

### 2.5. Precision ID Ancestry SNP Panel 

The Precision ID Ancestry SNP panel (Thermo Fisher Scientific, Waltham, MA, USA) was utilized for ancestry estimation to verify the anthropological assessment of European ancestry. PCR amplification was completed from 1 ng human DNA input with the following modifications to the standard protocol: 9 μL 2x KAPA HiFi HotStart Uracil+ ReadyMix, and 6 μL Precision ID Ancestry Panel. PCR was performed following the manufacturer’s protocol using 23 cycles. PCR-amplified libraries were purified using a 1.8x AMPure XP reaction (Beckman Coulter, Indianapolis, IN, USA), and eluted in 50 µL of Tris-EDTA. SNP amplification success was confirmed using the Agilent Bioanalyzer 2100 dsDNA HS kit. Library preparation of SNP amplicons was completed using the KAPA Hyper Prep kit (KAPA Biosystems, a Roche company, Wilmington, MA, USA) following the manufacturer’s protocol for sequencing on the Illumina MiSeq. DNA input into the library was determined using the Qubit 2.0 Fluorometer (Thermo Fisher Scientific). Adapter ligation utilized duplexed, 8 base pair adapters for Illumina (Integrated DNA Technologies, Skokie, IL, USA) at a concentration of 15 μM. Following adapter ligation, 8 PCR cycles were carried out for each sample, and amplified libraries were purified using a 0.8× AMPure XP reaction. DNA was re-suspended in 20 μL of Tris-EDTA. Library success was confirmed using the Agilent Bioanalyzer 2100 dsDNA HS kit. Samples and associated controls were pooled in equimolar concentration to generate a pool for sequencing. The molarity of the pool was determined using the Agilent Bioanalyzer 2100 dsDNA 7500 assay. The pool was diluted to a final loading concentration of 8 pM. The PhiX v3 Sequencing Control (Illumina, San Diego, CA, USA) was diluted and denatured separately, then spiked into the final pool at 5%. Paired-end sequencing was completed using an Illumina MiSeq Reagent Kit v3 (600-cycle, 2 × 300) on the MiSeq FGx Desktop Sequencer. 

The obtained raw fastq files were aligned to the human genome (Hg19) with a burrow-wheeler alignment algorithm—BWA-mem [10]. Samtools and Picardtools were used for sorting and indexing the BAM files [11,12], then Genome Analysis Toolkit (GATK) was used for variant calling and extracting information on base read counts per position [13]. The final genotypes, exceeding 6X coverage and minor allele frequency of 10%, were analyzed with three different methods for biogeographic ancestry (BGA) inference: Snipper [14], STRUCTURE/CLUMPAK [15,16] and principal component analysis (PCA). A reference population grid was gathered from publicly available curated data and was composed of 510 individuals in total, and divided into 6 main populations: Africa (AFR) 108 individuals—Yoruba in Ibadan, Nigeria; Europe (EUR) 99 individuals—Utah Residents (CEPH) with Northern and Western European Ancestry; East Asia (EAS) 103 individuals—Han Chinese in Beijing, China; America (AMR) 85 individuals—Peruvians from Lima, Peru; South Asia (SAS) 103 individuals—Gujarati Indian from Houston, Texas; and, Oceania (OCE) 14 individuals—Papuan from the Human Genome Diversity Project (HGDP) panel [17]. The reference data were extracted from 1000 Genomes Project Phase 3 (release 20130502) [18] for all populations but the Oceanians, which were extracted from the Simons Genome Diversity Project (SGDP) [19]. The gathered reference data took into account a balanced distribution of individuals per population, except for the Oceanians, which present in general a low number of samples with available data.

### 2.6. Y-STR Typing and Y-haplogroup Prediction 

Y-STR amplification was completed using the AmpFlSTR Yfiler PCR Amplification Kit (Applied Biosystems, Thermo Fisher Scientific), following a modified protocol intended for low copy number samples [20]. The targeted DNA input was 100–200 pg human DNA based on the Plexor HY quantification results. Amplified products were prepared for electrophoretic separation using the following conditions: 10 μL Hi-Di Formamide, 0.3 μL GeneScan 500 LIZ (LIZ-500) and 1.0 μL amplified product or allelic ladder. 

Data were analyzed using GeneMapper ID version 1.4 (Thermo Fisher Scientific), and allele calls were assigned using the allelic ladder provided by the manufacturer. An analytical threshold of 40 relative fluorescence units (RFU) and a stochastic threshold of 100 RFU were used for allele calling. Known artifacts (i.e., pull-up, spikes and split peaks resulting from incomplete adenylation) were manually removed based on results from previous studies [20]. A Y haplogroup assignment was determined from the consensus Y-STR profile using the Y haplogroup predictor NEVGEN [21]. To further resolve the R1b haplogroup, 4 variants representing major subclades of R1b with suspected Western European ancestry were typed: variant rs9786076 for R1b-L11, variant rs34276300 for R1b-P312, including additional sublineage branches of R1b-L21 using variant rs11799226, and R1b-Z195 using variant rs568477247 (see Table S1). Surname prediction was performed by searching for a match to the Y-STR profile within the FamilyTreeDNA website [20]. 

## 3. Results

### 3.1. DNA Quantification

The femoral sample of JB55 produced sufficient DNA for SNP and Y-STR typing based on the Plexor HY quantification (Table 1). Both the autosomal and Y-chromosomal targets were successfully amplified from both JB55 DNA extracts, indicating approximately 0.2 ng/µL autosomal DNA in a 200 µL DNA extract. Moderate degradation was observed, as twice as much DNA was amplified from the 99 bp autosomal target than was amplified from the 133 bp Y-chromosomal target. The reagent blanks failed to produce amplifiable human DNA.

### 3.2. Ancestry Estimation from Precision ID Ancestry SNP Panel

**Figure 3:**
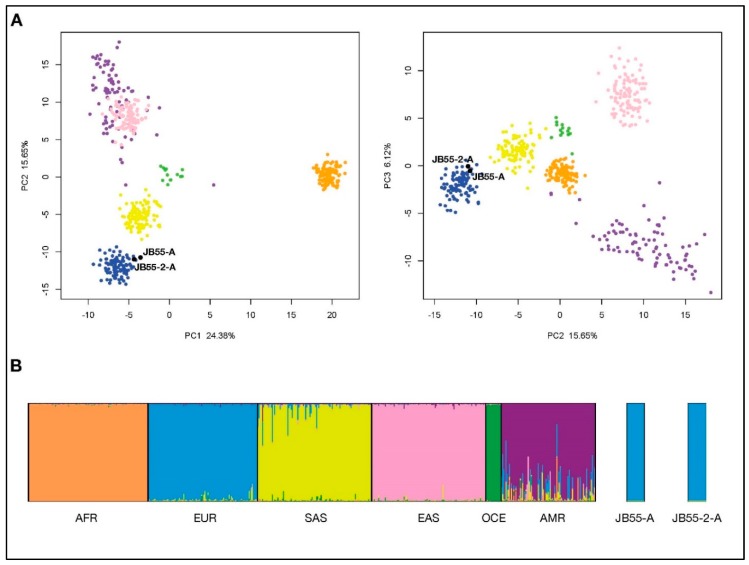
Bio-geographical ancestry inference for the two JB55 extracts using the Precision ID Ancestry single nucleotide polymorphism (SNP) panel and custom reference data. (**a**): Graphical representation of the principal components analysis (PCA) showing principal components (PC) 1 and 2 (left), and 2 and 3 (right). (**b**): Plot represents proportions of ancestry for K = 6 obtained with STRUCTURE. Individuals are colored in the PCA according to the six different clusters corresponding with six major populations in the STRUCTURE plot—AFR: Africa, EUR: Europe, SAS: South Asia, EAS: East Asia, OCE: Oceania and AMR: America.

A total of 87.8% and 89.7% of genotypes (considering the full 165 Precision ID Ancestry SNP Panel) were obtained for the two tested extracts, JB55-1 and JB55-2, respectively (Table S2). Genotypes were overall concordant between the two extracts in the successfully typed loci. The final genotypes were uploaded into Snipper with the reference population grid. According to the Snipper report, both extracts (JB55-1 and JB55-2) presented a predicted admixture of 100% European biogeographical ancestry and are more than a billion times more likely to be European than South Asian or American. PCA analysis (Figure 3A) reveals that both samples are found within the European cluster (in blue) when plotting the 1st component (PC1) versus the 2nd component (PC2) or the 2nd component (PC2) versus the 3rd component (PC3). Finally, a STRUCTURE run was performed for three simulations with K = 6 (previously established for the Precision ID Ancestry Panel as optimum K), for 100,000 burning and 100,000 Markov chain Monte Carlo iterations. STRUCTURE was run in correlated allele frequencies and admixed ancestry model and signed POPFLAG for reference populations. All three simulations produced the same patterns, which were combined and plotted using CLUMPAK (Figure 3B), showing both JB55-1 and JB55-2 belong to the European cluster (blue). When joining all analysis, we can infer both JB55 extracts are most probably of European descent. 

### 3.3. Y-STR Analysis

#### 3.3.1. Yfiler Y-STR Typing 

Complete Y-STR profiles were obtained from both JB55 DNA extracts, with the exception of DYS438, which was not typed in the second DNA extract (Table 2). To confirm the allele observed at DYS438, the Yfiler amplification product was sequenced on an Illumina MiSeq platform [22] using the methods described in Appendix A. The sequenced STR data (Tables S3 and S4) were analyzed using STRait Razor v.2.6 [23], which indicated a 12 allele at DYS438 in both of the JB55 DNA extracts (Table 3). Although the allelic read count was only 8 for the second JB55 DNA extract, the sequence data confirm the authenticity of the DYS438 allele having 12 repeats. 

#### 3.3.2. Y Haplogroup Estimation and Surname Prediction

The consensus Y-STR profile was then searched for in NEVGEN [21] for Y haplogroup estimation. The highest ranked result obtained was R1b, the most common Y haplogroup in Western Europe [24]. Further haplogroup resolution was gleaned from typing samples JB55-1 and JB55-2 using several Y-SNPs for the inference of successive R1b branches, R1b-L11, R1b-P312, R1b-L21 or R1b-Z195. For both samples, JB55 produced a derived allele at R1b-L11 (rs9786076-C), and at R1b-P312 (rs34276300-A), but yielded ancestral haplotypes for R1b-L21 (rs1179922-C) and R1b-Z195 (rs568477247-G). JB55 therefore could be resolved to the branch R1b-P312. With this information, the Y-STR profile was then compared against those in the publicly accessible FamilyTreeDNA R1b Project website [25] restricting to those individuals with the haplogroup R1b-P312 to identify any close matches that may have a “B” surname. Although surname prediction from Y-chromosomal DNA has limitations [26], it was worth attempting for JB55 due to the presumed initials on the coffin. The results yielded only two individuals in the R1b-P312 subclade who shared an almost identical Y-STR profile to JB55 with the exception of one locus (Y-GATA-H4), differing by only 1 repeat. JB55 has 12 repeats at Y-GATA-H4, whereas the two individuals in the FamilyTreeDNA R1b Project website have 11 repeats. The surname Barber was listed for both individuals, a compelling result given the expectation that the surname of JB55 also begins with a “B”.

## 4. Discussion

The JB55 DNA samples were of relatively high quality, given the age of this historical archaeology case dating to the mid-nineteenth century. Moderate DNA degradation was observed, yet ample DNA of sufficient fragment length was obtained for nuclear DNA profiling using forensic PCR amplification kits. This was demonstrated through the autosomal SNP analyses that showed JB55 to be of European ancestry. The genetic ancestry prediction is consistent with the anthropological assessment [1], and helps to establish the authenticity of the DNA results from this historical burial [1]. JB55’s Y-chromosomal DNA was analyzed using STR and SNP analyses, which indicated a R1b-P312 haplogroup that is common in Western Europe. The Y-STR profile, when searched for in a publicly accessible genetic genealogy website designated for haplogroup R1b, produced two close matches that both had the surname Barber. 

After discovering the predicted surname based on the Y-chromosomal DNA data, historical records were searched to determine whether there was a J. Barber buried in Griswold, Connecticut in the early 1800s. The Charles R. Hale Collection of Cemetery Inscriptions and Newspaper Notices, 1629–1934, contains a wealth of vital records that were documented for the state of Connecticut during the Works Progress Administration era [27]. A death notice in the Hale index describes a John Barber whose son Nathan Barber died in Griswold, CT in 1826 at the age of 12. This historical record closely matches the archaeological evidence, as a subadult “NB13” was discovered near JB55 in the cemetery, along with an adult female “IB” [1,4]. Although there is now a likely name for JB55, as well as NB, no further information on John Barber or Nathan Barber could be found in current genealogical databases or historical resources. A future project to compare DNA profiles between JB55, NB13 and IB is now in the planning stages. It may also be possible to test pathological rib samples from JB55 to evaluate the tentative diagnosis of tuberculosis. Future work involving genetic genealogy [28] may lead to living descendants of JB55, and possibly verify the identity of the Griswold, Connecticut vampire as John Barber.

To our knowledge, this is the first study that applies DNA testing to identify the remains of a historical case with no presumed identity. While JB55’s burial context offered some clues, the primary evidence that led to his (tentative) identification as John Barber was Y-chromosomal DNA profiling and surname prediction from a genealogical database. Thus, the JB55 case required a different approach than the traditional route taken to identify famous historical persons, such as King Richard III [29] and the Romanov family [30,31,32]. Since the presumed identities were known in these latter, high profile cases, DNA analysis was focused on the comparison of DNA profiles obtained from the unidentified remains with those of one or more living relatives. This was not possible for JB55, who was anonymous except for his initials, age, relative time period, and location. Similarly to the famous cases, however, the identification of JB55 was solved by making use of uniparentally inherited lineage markers, which are important when a generational gap precludes traditional means of kinship assessment from autosomal STR profiles. Together, these studies underscore the relevance of the haploid markers of mtDNA and the Y-chromosome in historical remains identification.

## Figures and Tables

**Table 1:** Plexor HY DNA quantification results from JB55 extracts 1 and 2, and corresponding reagent blanks (RBs). DNA concentration values are shown, and all DNA extracts contained 200 µL volume.

Sample ID	Autosomal DNA Concentration (99 bp target) (ng/µL)	Y-Chromosomal DNA Concentration (133 bp target) (ng/µL)	Autosomal: Y-Chromosomal DNA Ratio
JB55-1	0.2009	0.0871	2.3072
RB-1	0	0	0
JB55-2	0.1735	0.0802	2.1624
RB-2	0	0	0

**Table 2:** Y-chromosomal short tandem repeat (Y-STR) profiles obtained from each of the two JB55 DNA extracts using a low copy number AmpFISTR Yfiler amplification procedure and allelic separation by capillary electrophoresis.

Locus	JB55-1 Allele(s)	JB55-2 Allele(s)
DYS19	15	15
DYS385	11,13	11,13
DYS389I	13	13
DYS389II	29	29
DYS390	23	23
DYS391	11	11
DYS392	13	13
DYS393	13	13
DYS437	15	15
DYS438	12	No Data
DYS439	12	12
DYS448	19	19
DYS456	15	15
DYS458	17	17
DYS635	23	23
Y GATA H4	12	12

**Table 3:** DYS438 alleles obtained from Illumina sequencing of the Yfiler amplification product from the two JB55 DNA extracts. The stutter product with the highest read count is also shown (11 for JB55-1 and 13 for JB55-2).

JB55-1			JB55-2		
Allele	Read Count	% of Total Reads	Allele	Read Count	% of Total Reads
12	12,343	96.3%	12	8	88.9%
11	386	3.0%	13	1	11.1%
Other	87	0.7%	Other	0	0%
Total	12,816	100%	Total	9	100%

## Supplementary Materials

The following are available online at https://www.mdpi.com/2073-4425/10/9/636/s1, Table S1: Y-SNP typing of several R1b branches specific to Western European ancestry, Table S2: Genotype and number of reads per SNP for JB55-1 and JB55-2 samples, Table S3: JB55-1 sequenced Y-STR data obtained from STRait Razor v.2.6., Table S4: JB55-2 sequenced Y-STR data obtained from STRait Razor v.2.6.

## Appendix A. Illumina Sequencing of Yfiler Amplification Product

Yfiler amplicon product was purified using 1.8x Agencourt AMPure XP purification. Amplicon concentration for downstream library preparation was evaluated with the Agilent DNA High Sensitivity Kit on the 2100 BioAnalyzer instrument. Libraries were prepared using the KAPA Hyper Prep kit for Illumina platforms (Kapa Biosystems, Wilmington, MA, USA) following the manufacturer’s protocol for sequencing on the Illumina MiSeq. Adapter ligation utilized dual-indexed adapters for Illumina (Integrated DNA Technologies, Skokie, IL, USA) at a concentration of 15 μM. No library amplification was performed. Libraries were quantified using the Agilent DNA High Sensitivity Kit on the 2100 BioAnalyzer instrument. Based on the molarity, samples were individually normalized to 4 nM and pooled in equal volume for sequencing. The final pool was diluted to 10 pM and spiked with denatured PhiX Sequencing Control v3 at a 5% concentration. Single-end sequencing was completed for 500 cycles using an Illumina MiSeq Reagent Kit v3 (600-cycle) on the MiSeq FGx Desktop Sequencer. STRait razor v.2.6 [23] was used for sequenced STR data analysis.
